# The relationship between minute ventilation and end tidal CO_2_ in intubated and spontaneously breathing patients undergoing procedural sedation

**DOI:** 10.1371/journal.pone.0180187

**Published:** 2017-06-29

**Authors:** Jaideep H. Mehta, George W. Williams, Brian C. Harvey, Navneet K. Grewal, Edward E. George

**Affiliations:** 1 Department of Anesthesiology, The University of Texas Health Science Center at Houston, Houston, Texas, United States of America; 2 Respiratory Motion, Inc. Waltham, Massachusetts, United States of America; 3 Anesthesia, Critical Care, and Pain Medicine, Massachusetts General Hospital, Boston, Massachusetts, United States of America; National Yang-Ming University, TAIWAN

## Abstract

**Background:**

Monitoring respiratory status using end tidal CO_2_ (EtCO_2_), which reliably reflects arterial PaCO_2_ in intubated patients under general anesthesia, has often proven both inaccurate and inadequate when monitoring non-intubated and spontaneously breathing patients. This is particularly important in patients undergoing procedural sedation (e.g., endoscopy, colonoscopy). This can be undertaken in the operating theater, but is also often delivered outside the operating room by non-anesthesia providers. In this study we evaluated the ability for conventional EtCO_2_ monitoring to reflect changes in ventilation in non-intubated surgical patients undergoing monitored anesthesia care and compared and contrasted these findings to both intubated patients under general anesthesia and spontaneously breathing volunteers.

**Methods:**

Minute Ventilation (MV), tidal volume (TV), and respiratory rate (RR) were continuously collected from an impedance-based Respiratory Volume Monitor (RVM) simultaneously with capnography data in 160 patients from three patient groups: non-intubated surgical patients managed using spinal anesthesia and Procedural Sedation (n = 58); intubated surgical patients under General Anesthesia (n = 54); and spontaneously breathing Awake Volunteers (n = 48). EtCO_2_ instrument sensitivity was calculated for each patient as the slope of a Deming regression between corresponding measurements of EtCO_2_ and MV and expressed as angle from the x-axis (θ). All data are presented as mean ± SD unless otherwise indicated.

**Results:**

While, as expected, EtCO_2_ and MV measurements were negatively correlated in most patients, we found gross systematic differences across the three cohorts. In the General Anesthesia patients, small changes in MV resulted in large changes in EtCO_2_ (high sensitivity, θ = -83.6 ± 9.9°). In contrast, in the Awake Volunteers patients, large changes in MV resulted in insignificant changes in EtCO_2_ (low sensitivity, θ = -24.7 ± 19.7°, p < 0.0001 vs General Anesthesia). In the Procedural Sedation patients, EtCO_2_ sensitivity showed a bimodal distribution, with an approximately even split between patients showing high EtCO_2_ instrument sensitivity, similar to those under General Anesthesia, and patients with low EtCO_2_ instrument sensitivity, similar to the Awake Volunteers.

**Conclusions:**

When monitoring non-intubated patients undergoing procedural sedation, EtCO_2_ often provides inadequate instrument sensitivity when detecting changes in ventilation. This suggests that augmenting standard patient care with EtCO_2_ monitoring is a less than optimal solution for detecting changes in respiratory status in non-intubated patients. Instead, adding direct monitoring of MV with an RVM may be preferable for continuous assessment of adequacy of ventilation in non-intubated patients.

## Introduction

Whereas it is standard practice to both control and monitor ventilation during general anesthesia, it is equally important to monitor ventilation in non-intubated patients undergoing procedural sedation. End tidal CO_2_ (EtCO_2_) monitoring with capnography has become the standard of care in intubated patients for both confirming endotracheal tube placement and monitoring adequacy of ventilation [1,2]. Capnography with an endotracheal tube in place is considered a reliable method to non-invasively reflect arterial PaCO_2_ [3,4], however, measuring EtCO_2_ in spontaneously breathing patients can be inaccurate in certain settings, particularly during procedural sedation [5] and post-operatively in the post-anesthesia care unit [6–8]. Variables such as sensor positioning, changes in respiratory patterns, and changes in oxygen supplementation often distort EtCO_2_ measurements in non-intubated patients, rendering them unreliable. As a result, it is common for healthcare providers to overlook or discount information obtained from the capnography waveform [9].

Furthermore, since EtCO_2_ is an indirect indicator of respiratory status, it reflects ventilatory changes later than a direct measurement of ventilation, like minute ventilation (MV). This difference is especially important in spontaneously breathing subjects [10]. In current clinical applications, non-invasive measurements of EtCO_2_ do not allow health care practitioner to identify subtle changes in ventilation over a background of confounding factors such as ventilation/perfusion mismatch, partial airway obstruction, or metabolic derangement. For these reasons, capnography has never achieved wide clinical adoption in non-intubated patients [11].

Given the limitations of EtCO_2_ monitoring in precisely reflecting the respiratory status of patients, more emphasis may need to be placed on methods of volumetrically monitoring ventilation in non-intubated patients. With the introduction of a non-invasive Respiratory Volume Monitor (RVM) that can provide accurate measurements of MV, tidal volume (TV), and respiratory rate (RR) in non-intubated patients [12,13], direct monitoring of ventilation in non-intubated patients has become available both inside and out of the operating room. Here we studied the ability for conventional EtCO_2_ monitoring to reflect changes in ventilation in non-intubated surgical patients undergoing spinal anesthesia and procedural sedation. We computed the instrument sensitivity of a EtCO_2_ monitor when detecting changes in MV in these non-intubated surgical patients and compared and contrasted this sensitivity to both intubated patients under general anesthesia and spontaneously breathing volunteers.

## Materials and methods

### Experimental design

Continuous respiratory data (MV, TV, and RR) were collected from an impedance-based RVM (ExSpiron, Respiratory Motion, Inc., Waltham, MA) simultaneously with capnography data (EtCO_2_) in patients in three groups: patients under Procedural Sedation (1), patients under General Anesthesia (2), and Awake Volunteers (3). The Procedural Sedation group was of primary interest in this manuscript and the other two groups were used effectively as “control” groups providing limiting conditions based on the level of sedation ranging from “none” in the Awake Volunteers to “deep” in the intubated and mechanically-ventilated patients in the General Anesthesia group.

#### Procedural Sedation cohort

In this group, patients underwent elective joint replacement surgery with spinal anesthesia and procedural sedation. EtCO_2_ data were collected from a sampling nasal cannula with oral scoop sampling port (Covidien Smart CapnoLine Plus Oral/Nasal, Boulder, CO) using a ventilator (Dräger Apollo, Andover, MA). Anesthesia was initiated immediately prior to surgery, typically with an intrathecal dose of bupivacaine 0.5% (1.5–4.0 ml) and supplemented with midazolam, propofol, and fentanyl for sedation. Additional intraoperative opioids such as hydromorphone were rarely used in Spinal Anaesthesia cases. Typically, patients undergoing knee surgery also received a femoral nerve block, consisting of either 20 ml ropivacaine 0.2% or 20 ml bupivacaine 0.25%, administered in preoperative holding.

#### General Anesthesia cohort

In this group, patients underwent elective joint replacement surgery under general anesthesia. MV and EtCO_2_ data were collected from the endotracheal tube using a ventilator (Dräger Apollo, Andover, MA). Anesthesia was initiated immediately prior to surgery, with various doses of a muscle relaxant (rocuronium, vecuronium, or cisatracurium), in conjunction with sedatives (midazolam, propofol, and ketamine), and opioids (fentanyl, hydromorphone, meperidine, remifentanil, and morphine). Typically, patients undergoing knee surgery also received a femoral nerve block, consisting of either 20 ml ropivacaine 0.2% or 20 ml bupivacaine 0.25%, administered in preoperative holding. A detailed summary of relevant medications used intra-operatively in the Procedural Sedation and General Anaesthesia cohorts, with frequency and dosage, can be found in Table 1.

**Table 1:** Intraoperative medications in the Procedural Sedation and General Anaesthesia cohorts.

	PROCEDURAL SEDATION N = 58				GENERAL ANESTHESIA N = 54			
			Dose				Dose	
	N		Mean	SD	N		Mean	SD
**Spinal:**	**58**	**100%**	** **	** **	**0**	**0%**	** **	** **
Bupivacaine 0.5% (ITHEC) (ml)	54	93%	2.8	0.8	0	0%		
Bupivacaine 0.75% (ITHEC) (ml)	4	7%	2.0	0.5	0	0%		
**Paralytic:**	**0**	**0%**			**42**	**78%**		
Rocuronium (mg)	0	0%			21	39%	68.2	28.8
Cisatracurium (mg)	0	0%			14	26%	15.7	6.7
Vecuronium (mg)	0	0%			6	11%	12.3	4.1
*LMA Insertion (no paralytic)*	0	0%			*4*	7%		
*Paralytic not specified*	0	0%			*1*	2%		
**Inhalation Agent:**	**0**	**0%**			**51**	**94%**		
Sevoflurane	0	0%			43	80%		
Isoflurane	0	0%			8	15%		
**Reversal Agent:**	**0**	**0%**			**31**	**57%**		
Neostigmine (mg)	0	0%			31	57%	2.8	1.1
**Femoral Block:**	**36**	**62%**			**22**	**41%**		
Ropivacaine 0.2% (ml)	29	50%	19.7	1.3	20	37%	21.0	4.5
Bupivacaine 0.25% (ml)	8	14%	24.5	14.4	2	4%	17.5	3.5
**Sedatives:**	**57**	**98%**	** **	** **	**54**	**100%**		
Midazolam (mg)	54	93%	2.5	1.1	44	81%	2.1	0.9
Propofol (Total) (mg)	48	83%	331	252.3	54	100%	281	300.2
:: *bolus (mg)*	11	19%	32.1	20.7	54	100%	214	74.5
:: *infusion (mg)*	45	78%	345	245.4	3	6%	1196	530.6
Ketamine (mg)	1	2%	46.4		3	6%	158	94.6
**Opioids:**	**51**	**88%**	** **	** **	**54**	**100%**		
Fentanyl (mcg)	51	88%	98.4	51.1	52	96%	222	85
Hydromorphone (mg)	2	3%	0.5	0.0	44	81%	1.3	1.0
Meperidine (mg)	0	0%			1	2%	25.0	0
Remifentanil (mg)	0	0%			3	6%	0.4	0.2
Morphine (mg)	0	0%			4	7%	8.3	3.5
**Other:**						** **		
Haloperidol	2	3%	1.0	0.0	28	52%	1.0	0.0
Succinylcholine	0	0%			10	19%	114	50

#### Awake Volunteers cohort

In this group, spontaneously breathing subjects performed a total of six breathing trials at varying prescribed respiratory rates for a total of 13 min. In the first and last trials, subjects were instructed to breathe normally, while in the middle four trials, subjects alternated between fast (25 bpm) and slow (5 bpm) as set by a metronome. EtCO_2_ data were collected from a sampling nasal cannula with oral scoop sampling port (Covidien Smart CapnoLine Plus Oral/Nasal, Boulder, CO) using a dedicated capnograph (Capnostream 20, Covidien, Boulder, CO). All subjects responded to an Institutional Review Board-approved advertisement.

#### Equipment

In all three cohorts, the RVM collected bio-impedance traces via an electrode padset placed in the recommended positions: sternal notch, xiphoid, and right mid-axillary line at the level of the xiphoid. The skin was prepped and the padset applied in a fashion similar to that used in standard ECG electrode placement. At the beginning of the study, the RVM was calibrated against a ventilator in the General Anesthesia group, a Wright sprirometer (Mark 14, nSpire Health, Inc., Longmont, CO) in the Procedural Sedation group, and a heated pneumoatchometer (Heated FVL, Morgan Scientific, Haverhill, MA) in the Awake Volunteers group.

#### Institutional review board and consent

Inclusion criteria for the all three cohorts were English-speaking men and women aged 18 years to 99 years. Exclusion criteria for the Procedural Sedation and General Anesthesia groups were pregnant females, patients with an electronic implantable device, and surgery positions other than supine or lateral. Exclusion criteria for the Awake Volunteers group were hospitalization within 30 days before the study and pregnant females.

The study for the Procedural Sedation and General Anesthesia cohorts was approved by the Partners Institutional Review Board, Boston, MA (2011P002898). The study for the Awake Volunteers group was approved by the Schulman Associates Institutional Review Board, Cincinnati, OH (201102306). All patients gave informed written informed consent.

### Data and statistical analysis

The ability of EtCO_2_ to reflect changes in MV (instrument sensitivity) was calculated for each patient. Specifically, instrument sensitivity was defined as the slope calculated by a Deming regression between individual corresponding measurements of EtCO_2_ and MV. The slopes of the regression were presented as angles from the x-axis ($\theta =\tan^{-1}\left(\frac{\Delta EtCO_{2}}{\Delta MV}\right)$). A steep correlation line (i.e., θ ≈ -90°) corresponds to high instrument sensitivity, indicating a small change in MV leads to a large change in EtCO_2_. A flatter correlation line (i.e., θ ≈ 0°) corresponds to low instrument sensitivity, indicating a small change in MV results in almost no change in EtCO_2_. The stated EtCO_2_ accuracies of the ventilator and capnograph used in this study were ±3.8 mm Hg [14] and ±2.0 mm Hg [15], respectively. Previous work suggests that the minimally acceptable instrument sensitivity for clinically-relevant EtCO_2_ monitoring is -4.0 ^mmHg^/_L/min_ (i.e., θ = -76°) [10]. For each patient, MV was calculated as a percent of their individual predicted MV (MV_PRED_), based on each patient’s body surface area and sex[16,17], which has been shown to be a better predictor of actually observed MV during spontaneous respiration than MV_PRED_ based on Ideal Body Weight (IBW) [18]. Unbalanced one-way ANOVAs were used to compare demographics, instrument sensitivities, as well as average EtCO_2_ and MV measurements across cohorts. All data are presented as mean ± SD unless otherwise indicated.

## Results

Data were collected from 160 patients across the three cohorts (Table 2). Height and weight were not significantly different across the three cohorts (p = 0.12 and p = 0.17, respectively), however, BMI and age were significantly lower in the Awake Volunteers group compared to the Procedural Sedation and General Anesthesia groups (BMI: p < 0.02; age: p < 0.0001 for both comparisons). Procedural Sedation and General Anesthesia patients tended to have more comorbidities than the Awake Volunteers. All patients in the Procedural Sedation and General Anesthesia groups received supplemental oxygen and both groups had a similar average FiO_2_ delivered throughout the procedure (p = 0.86).

**Table 2:** Subject cohort anthropometrics and comorbidities.

Anthropometrics	Procedural Sedation	General Anesthesia	Awake Volunteers
**Number of Patients**	58	54	48
**Males / Females**	30/28	23/31	33/15
**Age (SD), years**	69.1 (9.0)	65.2 (12.1)	45.7 (13.6)
**Weight (SD), kg**	89.8 (16.7)	88.4 (19.1)	81.5 (22.8)
**Height (SD), cm**	170.7 (10.9)	168.0 (9.1)	171.7 (10.4)
**BMI (SD), kg/m**^**2**^	30.9 (5.5)	31.3 (6.2)	27.3 (6.1)
**Body Surface Area (SD), m**^**2**^	1.97 (0.22)	2.01 (0.21)	1.93 (0.29)
**ASA Physical Status, 1/2/3**	2/42/14	3/29/21	
**Obstructive Sleep Apnea (%)**	5 (8.6)	6 (11.1)	4 (8.3)
**Asthma (%)**	12 (20.7)	5 (9.3)	4 (8.3)
**Coronary Artery Disease (%)**	5 (8.6)	6 (11.1)	0 (0.0)
**Chronic Obstructive Pulmonary Disease (%)**	3 (5.2)	4 (7.4)	1 (2.1)
**Length of Monitoring (SD), min**	77 (38)	149 (73)	13 (0)
**Joint Replaced (Knee / Hip)**	36/18	28/26	
**Supplemental O**_**2**_**, Average FiO**_**2**_ **(SD), %**	51.5 (13.1)	51.1 (10.3)	0 (0)

Patients in the three cohorts had similar MV_PRED_ (p = 0.25, Table 3). During the course of the surgical procedure, General Anesthesia patients had an average MV of 80.9% MV_PRED_, suggesting a decreased metabolic function resulting from the anesthesia. In contrast, the Procedural Sedation patients had a significant higher average MV of 148.4% MV_PRED_, due to both an increased TV and RR (p < 0.0001), indicative of their lightly sedated state. In comparison, during normal breathing trials, Awake Volunteers maintained close to their predicted MV (106.3% MV_PRED_).

**Table 3:** Subject cohort respiratory metrics.

Respiratory Metric	Procedural Sedation	General Anesthesia	Awake Volunteers
**Predicted Minute Ventilation, MV**_**PRED**_ **(SEM), L/min**	7.0 (0.1)	6.9 (0.1)	6.8 (0.1)
**Minute Ventilation (SEM), L/min**	10.3 (0.7)	5.6 (0.2)	7.1 (0.4)
**Minute Ventilation (SEM), % MV**_**PRED**_	148.4 (8.5)	80.9 (2.3)	106.3 (6.1)
**Tidal Volume (SEM), mL**	673 (39)	481 (13)	623 (39)
**Respiratory Rate (SEM), bpm**	15.2 (0.3)	11.6 (0.3)	12.4 (0.6)

**Fig 1:**
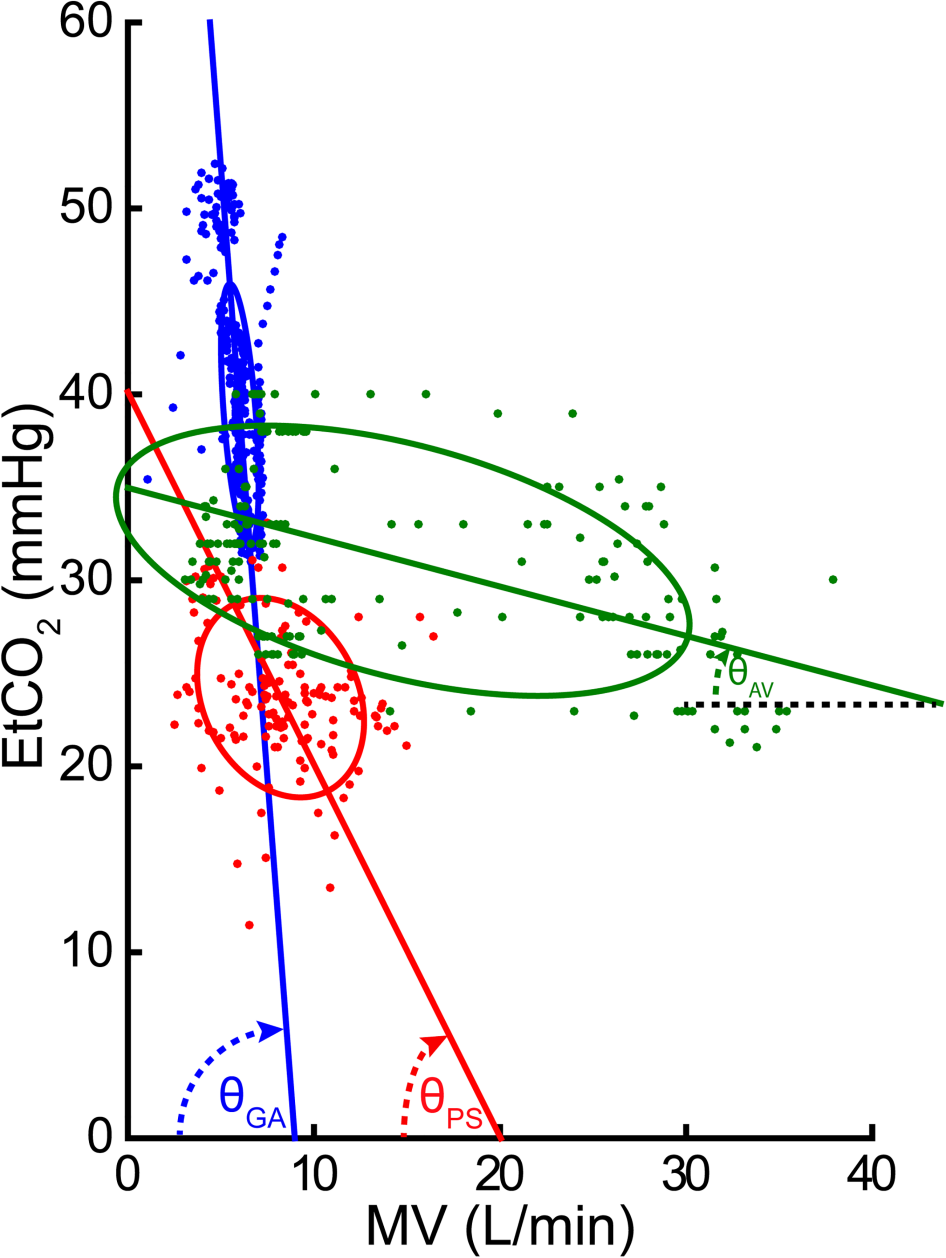
Representative correlations between MV and EtCO_2_. Data from three individual patients are included, one from each group: Procedural Sedation (red), General Anesthesia (blue) and Awake Volunteer (green). Each data point corresponds to a single 30 sec measurement pair (MV and EtCO_2_). The lines (Deming regressions) and confidence ellipses (±1 SD) show the best-fits to the data. In a representative patient from the General Anesthesia cohort, a 1 L/min increase in MV resulted in a 13.2 mmHg decrease in EtCO_2_ (EtCO_2_ instrument sensitivity (i.e., slope) = 13.2 mmHg/L/min = θ_GA_ = -85.7°). In a patient from the Awake Volunteers group, across a range of breathing patterns, a 1 L/min increase in MV resulted in a 0.27 mmHg decrease in EtCO_2_ (EtCO_2_ instrument sensitivity = 0.27 mmHg/L/min = θ_AV_ = -14.9°). The patient from the Procedural Sedation group falls between the patients from General Anesthesia and Awake Volunteers groups. Specifically, a 1 L/min increase in MV led to 2.0 mmHg decrease in EtCO_2_ (EtCO_2_ instrument sensitivity = 2.0 ^mmHg^/_L/min_, corresponding with θ_PS_ = -63.5°).

In a given patient, a plot of EtCO_2_ measurements against corresponding MV measurements produced a negatively correlated distribution: as MV increased, EtCO_2_ generally decreased (Fig 1). In a representative intubated patient under General Anesthesia (blue), small changes in MV (from 5.3 to 6.7 L/min) triggered large changes in EtCO_2_ (from 43.7 to 34.1 mmHg). Specifically, a 1 L/min increase in MV resulted in a 13.2 mmHg decrease in EtCO_2_, yielding a high EtCO_2_ instrument sensitivity of 13.2 mmHg/L/min, equivalent to θ_GA_ = -85.7° (nearly vertical line, as shown in Fig 1). In contrast, in an Awake Volunteer (green), a ten times larger change in MV (from 4.3 to 25.2 L/min) was required to trigger a similar change in EtCO_2_ (from 26.1 to 36.0 mmHg). For this patient, a 1 L/min increase in MV resulted in a 0.27 mmHg decrease in EtCO_2_, yielding a low EtCO_2_ instrument sensitivity of 0.27 mmHg/L/min, equivalent to θ_AV_ = -14.9° (nearly horizontal line, as shown in Fig 1). Interestingly, patients under Procedural Sedation (red), fell between the General Anesthesia and Awake Volunteer patients. Specifically, in the example patient, a 1 L/min increase in MV led to 2.0 mmHg decrease in EtCO_2_, yielding an instrument sensitivity of 2.0 mmHg/L/min, equivalent to θ_PS_ = -63.5°.

**Fig 2:**
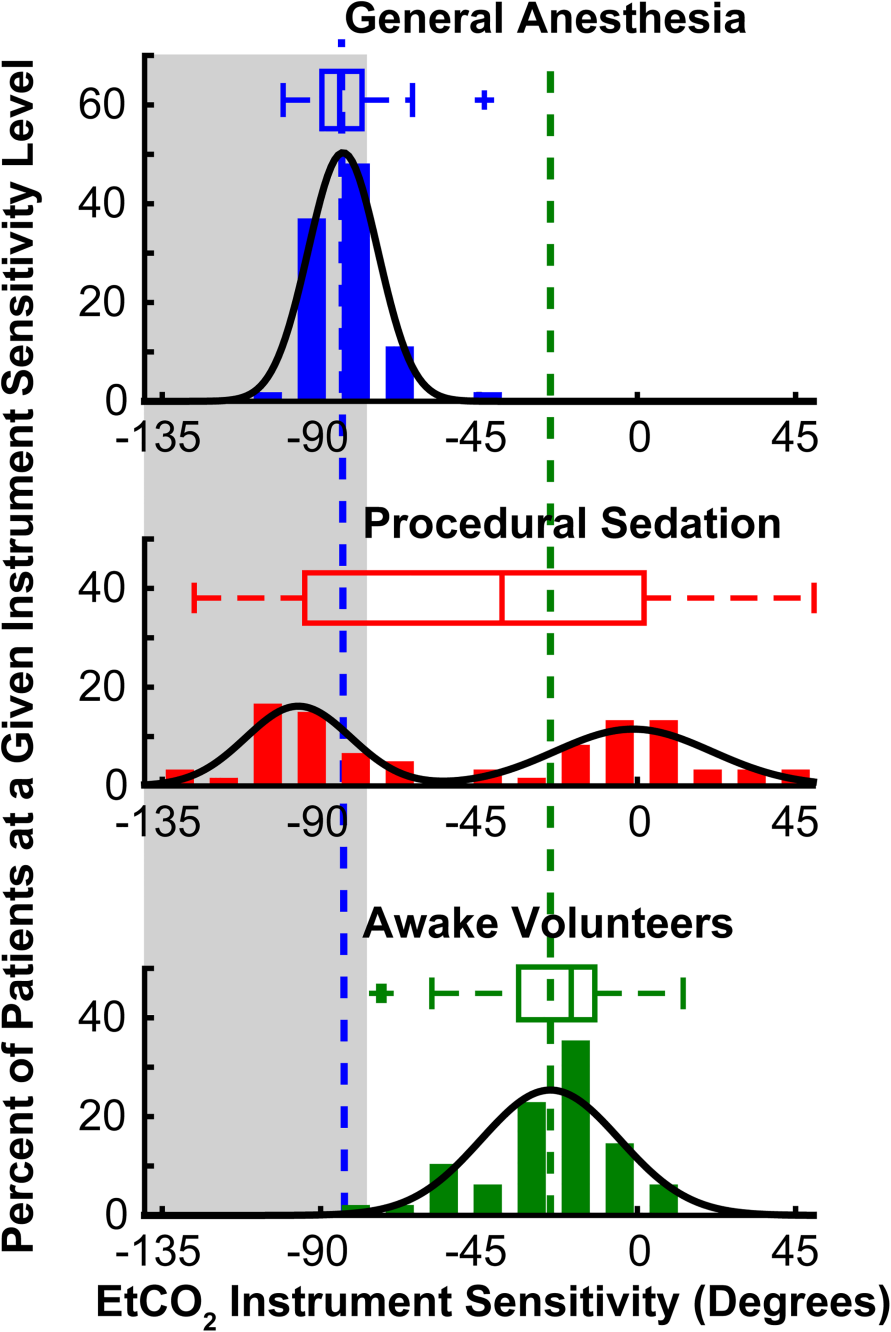
Distributions of EtCO_2_ instrument sensitivity to changes in MV. For each patient group (General Anesthesia (top, blue), Procedural Sedation (middle, red), and Awake Volunteers (bottom, green)), the distribution of EtCO_2_ instrument sensitivity is presented as both a box-plot and histogram. Each box-plot shows the median EtCO_2_ instrument sensitivity (middle vertical line), the box extends from the 25th to 75th percentile, the whiskers extend to the most extreme non-outlier data points, and statistical outliers are plotted individually (plus signs). The median EtCO_2_ instrument sensitivities were -85.1°, -38.1°, and -20.2° for the General Anesthesia, Procedural Sedation, and Awake Vounteer cohorts, respectively. The General Anesthesia and Awake Volunteer cohorts had unimodal distributions of EtCO_2_ instrument sensitivity and single normal distributions were fit to these data (black lines). EtCO_2_ instrument sensitivities were significantly higher in the intubated patients under General Anesthesia (θ = -83.6 ± 9.9°, vertical dashed blue line) compared to non-intubated Awake Volunteers over a range of prescribed breathing patterns (θ = -24.7 ± 19.7°, vertical dashed green line, p < 0.0001). The distribution of EtCO_2_ instrument sensitivity for the Procedural Sedation cohort was bimodal. Therefore, a mixture of two normal distributions was therefore fit to these data. Approximately half of the patients experienced high EtCO_2_ instrument sensitivity (θ = -96.6 ± 15.0°), consistent with the General Anesthesia patients, while the remaining patients had low instrument sensitivity (θ = -1.2 ± 22.4°), consistent with the awake volunteers. Clinically-relevant EtCO_2_ is indicated by the shaded gray area. The majority of General Anesthesia patients (43/54, 80%) had clinically-relevant EtCO_2_ instrument sensitivity. In contrast, less than half of Procedural Sedation patients (24/58, 41%) and no patients in the Awake Volunteer cohort demonstrated clinically-relevant EtCO_2_ instrument sensitivity of -76°.

EtCO_2_ instrument sensitivity was calculated for each patient and the distributions of the instrument sensitivities for each of the three groups were analyzed (Fig 2). The median EtCO_2_ instrument sensitivities were -85.1°, -38.1°, and -20.2° for the General Anesthesia, Procedural Sedation, and Awake Vounteer cohorts, respectively. The General Anesthesia and Awake Volunteers cohorts had unimodal distributions of EtCO_2_ instrument sensitivity, well described by single Gaussian functions fit to these data. EtCO_2_ instrument sensitivities were significantly higher in the intubated patients under General Anesthesia (θ = -83.6 ± 9.9°) compared to non-intubated Awake Volunteers (θ = -24.7 ± 19.7°, p < 0.0001). Interestingly, the distribution of EtCO_2_ instrument sensitivity for the Procedural Sedation cohort was clearly bimodal, illustrating a lack of uniformity in this group. A mixed-model of two Gaussian was therefore fit to these data, showing that 47% (27/54) of the patients experienced high EtCO_2_ instrument sensitivity (θ = -96.6 ± 15.0°), similar to the General Anesthesia patients, while the remaining patients had low instrument sensitivity (θ = -1.2 ± 22.4°), similar to the Awake Volunteers. Importantly, while the majority of patients in the General Anesthesia group (43/54, 80%) had EtCO_2_ instrument sensitivity which showed changes in EtCO_2_ in the clinically-relevant range (shaded gray area), less than half of Procedural Sedation patients (24/58, 41%) and no patients in the Awake Volunteers cohort had clinically-relevant EtCO_2_ instrument sensitivity of -76° corresponding to -4 ^mmHg^/_L/min_.

**Fig 3:**
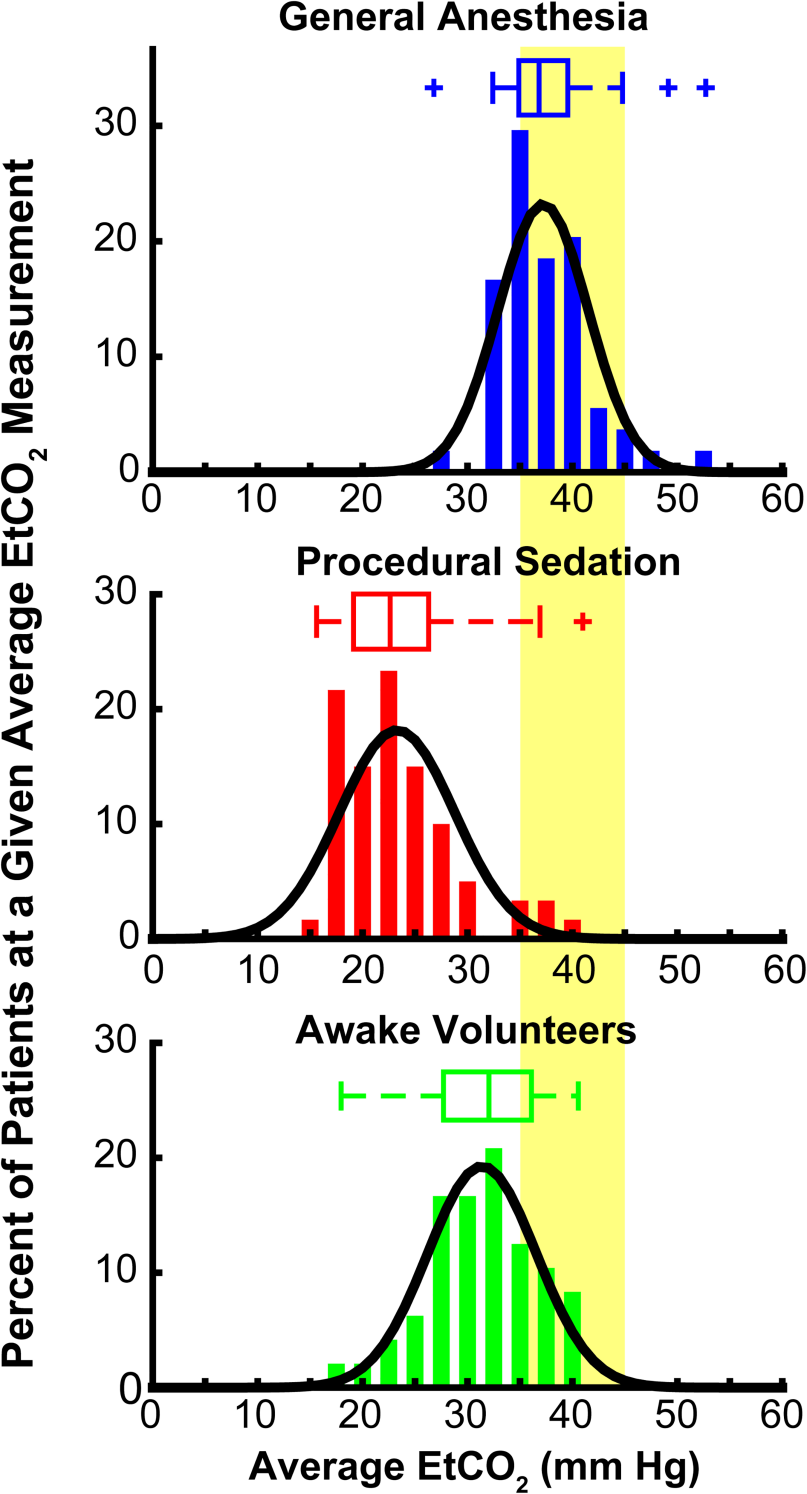
Distributions of average EtCO_2_ measurements. For each patient group (General Anesthesia (top, blue), Procedural Sedation (middle, red), and Awake Volunteers (bottom, green)), the distribution of average EtCO_2_ is presented as both a histogram and box-plot. Each box-plot shows the median EtCO_2_ instrument sensitivity (middle vertical line), the box extends from the 25th to 75th percentile, the whiskers extend to the most extreme non-outlier data points, and statistical outliers are plotted individually (plus signs). EtCO_2_ values for all three groups were unimodal and a single normal distribution was fit to each group (black lines). The average EtCO_2_ in the General Anesthesia cohort (37.2 ± 4.3 mmHg) was significantly higher than in the Awake Volunteers (31.4 ± 5.2 mmHg) which in turn was higher than the Procedural Sedation groups (23.3 ± 4.8 mmHg) (p < 0.0001). The normal range of EtCO_2_ (35–45 mmHg) is indicated by the shaded yellow area. The majority of General Anesthesia patients (37/54, 69%) had an average EtCO_2_ measurement within this normal range. In contrast, only 27% (13/48) of Awake Volunteers across the range of prescribed respiratory patterns and 5% (3/58) of Procedural Sedation patients had an average EtCO_2_ measurement within the normal range.

The average EtCO_2_ measurement over the length of monitoring was calculated for each patient and the distributions of these average measurements for the three groups were analyzed (Fig 3). Measured EtCO_2_ values were higher in the General Anesthesia (37.2 ± 4.3 mmHg) group than the Procedural Sedation (23.3 ± 4.8 mmHg) and Awake Volunteers (31.4 ± 5.2 mmHg) groups (p < 0.0001). Interestingly, the average measured EtCO_2_ of the Awake Volunteers across the prescribed breathing patterns was higher than the Procedural Sedation group (p<0.0001). The majority of General Anesthesia patients (37/54, 69%) had an average EtCO_2_ measurement within the normal range of EtCO_2_ (35–45 mmHg, shaded yellow area). In contrast, only 27% (13/48) of the Awake Volunteers and only 5% (3/58) of the Procedural Sedation patients had an average EtCO_2_ measurement within the normal range.

## Discussion

In this study, we assessed and quantified the ability of capnography to measure and reflect real-time changes in respiratory status, specifically ventilation (MV), in non-intubated patients undergoing procedural sedation. First, we quantified EtCO_2_ instrument sensitivity for each patient as the slope of a Deming regression between corresponding measurements of EtCO_2_ and MV. Next, we compared the instrument sensitivity between patients under Procedural Sedation and two control groups: General Anesthesia and Awake Volunteers. In the intubated patient under General Anesthesia, we found a strong relationship between MV and EtCO_2_ (median EtCO_2_ instrument sensitivity of -85.1°). This EtCO_2_ instrument sensitivity was better than the clinically-relevant EtCO_2_ instrument sensitivity of -76°, confirming EtCO_2_ measurements in intubated patients could adequately reflect changes in MV. In contrast, in the non-intubated patients (i.e., both the Procedural Sedation and Awake Volunteer groups), the relationship between MV and EtCO_2_ is much weaker (median EtCO_2_ instrument sensitivities of -38.1° and -20.2°, respectively) and better than the clinically-relevant instrument sensitivity of -76° in only 23% (24/106) of the non-intubated patients. This finding indicates that the EtCO_2_ instrument sensitivity in non-intubated and spontaneously breathing individuals may not be adequate for detecting meaningful changes in MV in over three-fourth of patients.

We found EtCO_2_ instrument sensitivity in Procedural Sedation patients exhibited a bimodal distribution with approximately half of the patients having high EtCO_2_ instrument sensitivity and other half exhibiting low EtCO_2_ instrument sensitivity. We saw very similar low EtCO_2_ instrument sensitivity to that demonstrated in the Awake Volunteer cohort. One potential explanation for this observation is that more deeply sedated Procedural Sedation patients behaved similarly to patients under General Anesthesia and therefore showed high EtCO_2_ instrument sensitivity, while less deeply sedated Procedural Sedation patients behaved more similarly to the Awake Volunteers and maintained their ability to modulate MV in response to changes in EtCO_2_. Within the Procedural Sedation group, there was not a significant difference between average supplemental oxygen FiO_2_ delivered to patients with high EtCO_2_ instrument sensitivity compared to patients with low EtCO_2_ instrument sensitivity (p = 0.70).

In spontaneously breathing patients, an increase in partial pressure of carbon dioxide in the arterial blood (PaCO_2_) triggers an increase in ventilation in order to maintain a relatively constant level of PaCO_2_ within the physiological range. In spontanously breathing patients under anesthesia, this respiratory drive to increase ventilation in response to hypercapnia is blunted through depressed drive from both central and peripheral muscular chemoreceptors [19]. These decreases in MV results in a buildup of PaCO_2_, and in turn EtCO_2_, without a compensatory increase in ventilation.

In mechanically ventilated patients, measurements of EtCO_2_ provide a clinically useful surrogate for the PaCO_2_, and capnography is the standard of care in this setting [3,4]. Recently, capnography was proposed as a tool to detect respiratory depression in non-intubated patients earlier than pulse oximetry [20–23]. However, despite the initial enthusiasm, capnography has not achieved wide clinical adoption in hospital settings such as the post-anesthesia care unit and general hospital floor [6,11]. Even in the more controlled setting of the procedure room, capnography has proved to be less reliable than anticipated due to cannula dislodgement, patient noncompliance, and complexity in interpreting CO_2_ waveforms [9,11,24]. Other factors which decrease the accuracy and utility of capnography in non-intubated patients include mouth versus nose breathing, changes in flow of oxygen, procedures requiring oral intervention or lack of access to the head of the bed to ensure proper cannula placement [25]. Furthermore, EtCO_2_ does not consistently reflect PaCO_2_, particularly in patients with cardiac and respiratory failure and in patients with a high ventilation-perfusion ratio [6–8,26–29]. Even when reliable EtCO_2_ measurements are obtained, they provide a lagging indicator of respiratory performance rather than direct measure of changes in respiratory volumes [30].

Recent work has shown that capnography has poor instrument sensitivity to changes in MV in a cohort of spontaneously breathing volunteers [10]. Here we evaluated the capability of capnography to detect changes in respiratory status in a group of patients undergoing procedural sedation for a surgical procedure and demonstrated the surprisingly variable instrument sensitivity of capnography, often outside of the clinically-relevant range. The data suggest that the RVM can provide more clinically useful information than capnography during procedural sedation. These results are in-line with the findings that the RVM provides an indication of respiratory depression in advance of changes in pulse oximetry in patients following orthopedic procedures [31] and can also be used to identify and quantify respiratory depression following the administration of midazolam peri-operatively [32]. Furthermore, Holley et al. demonstrated the superiority of MV moniting over monitoring RR alone during procedural sedation for upper endoscopic procedures [33]. During these procedures, the RVM detected decreases in MV in response to sedatives and also identified increases in MV following airway maneuvers such as chin lifts and jaw thrusts [34,35]. In addition to changes in MV, the RVM is also able to detect periods of airway obstruction [12]. Combining our findings with these previous reports supports the conclusion that monitoring respiratory volumes directly in non-intubated patients under procedural sedation delivers earlier and more reliable assessment of respiratory status than capnography or pulse oximetry, providing a better alternative for use in adjusting sedation to maintain both patient safety and comfort.

The most challenging aspect of this study was the establishment of clinically-relevant instrument sensitivity in the Awake Volunteers control group. Whereas the General Anesthesia cohort was very similar to the Procedural Sedation cohort, the Awake Volunteers were in some ways out of place in this study. In both Procedural Sedation and General Anesthesia groups all measurements of EtCO_2_ and MV were done in a purely observational manner, the Awake Volunteers had to be instructed to breathe over a range of breathing patterns in order to provide a wide measurement range over which EtCO_2_ instrument sensitivity could be accurately estimated. The Awake Volunteers were also younger, had lower BMIs, and fewer comormidites than the General Anesthesia and Procedural Sedation patients. Therefore, the combination of these factors suggest that the capacity for exhalation of CO_2_ is likely less in the General Anesthesia and Procedural Sedation cohorts compared to the Awake Volunteers group. Nonetheless, the addition of this cohort provided us with data from non-anesthetized patients to help bracket and better understand the group of lightly sedated Procedural Sedation patients. Interestingly, it also helped to profile the fact that in general, the Procedural Sedation group was hyperventilating, with an average MV of 148% MV_PRED_.

There are several other limitations to this study. First, the anesthetic and sedation regimens were chosen by the anesthesiologist and individualized for each patient. The level of sedation in the Procedural Sedation cohort was not standardized. Since individual patients respond significantly differently to similar levels of opioids and sedatives, even with a standardized sedation protocol, sedation levels still might vary greatly from patient to patient. Depth of anesthesia was not monitored with a bispectral monitor or with sedation scores as part of the standard of care. Future studies where depth of anaesthesia is also monitored are needed to test if the bimodal distribution of EtCO_2_ instrument sensitivies is explained by sedation level.

## Conclusions

While EtCO_2_ is a useful indicator of respiratory status in patients under General Anesthesia, its sensitivity to changes in ventilation is greatly reduced in non-intubated patients. Therefore, agumenting standard patient care with EtCO_2_ monitoring is a suboptimal solution for monitoring respiratory status in non-intubated patients undergoing Procedural Sedation. The addition of direct monitoring of MV with an RVM may be preferable for primary continuous assessment of adequate ventilation of non-intubated patients undergoing procedural sedation.
